# Deacetylation of SOD3 by sirtuins restores furin cleavage

**DOI:** 10.1016/j.rbc.2025.100062

**Published:** 2025-10-24

**Authors:** Emily C. Mitchem, Peter S. Harris, Cole R. Michel, Courtney D. McGinnis, Shashikant Ray, Krishna M.G. Mallela, James R. Roede, Steen V. Petersen, Eva S. Nozik, Kristofer S. Fritz

**Affiliations:** a Skaggs School of Pharmacy and Pharmaceutical Sciences, University of Colorado Anschutz Medical Campus, Aurora, CO, 80045, USA; b Cardiovascular Pulmonary Research Laboratories and Pediatric Critical Care, Department of Pediatrics, The University of Colorado Anschutz Medical Center, Aurora, CO, 80045, USA; c Department of Biotechnology, Mahatma Gandhi Central University, Motihari, Bihar, India, 845401; d Department of Biomedicine, Aarhus University, Aarhus, Denmark

**Keywords:** Lysine acetylation, Sirtuin, Oxidative stress, Superoxide dismutase 3, EC-SOD, Furin

## Abstract

Accumulation of superoxide radicals leads to disrupted redox signaling and oxidative damage. The primary extracellular scavenger of superoxide is extracellular superoxide dismutase (SOD3), a crucial enzyme in maintaining antioxidant status and proper immune function. SOD3 distribution to the extracellular matrix is determined by the presence of a C-terminal heparin-binding domain (HBD). This region can be removed through intracellular proteolytic processing by furin. Cleavage of the HBD has been shown to be modulated by post-translational cysteine redox status, regulating the secretion of SOD3. Interestingly, other members of the SOD family, SOD1 and SOD2, are known to be inhibited by lysine acetylation, a metabolically linked post-translational modification (PTM) that can alter protein structure, function, and localization. Yet, no reports describe the effect of acetylation on SOD3. Here, immunoblotting and mass spectrometry (MS) were used to quantify the global and site-specific acetylation of recombinant human SOD3. Interestingly, a predicted and targeted parallel reaction monitoring (PRM) MS-based approach was necessary to identify lysine acetylation within the C-terminal HBD of SOD3. Acetylation was found to prevent furin cleavage with no impact on SOD3 activity. Our results also reveal that SOD3 is robustly deacetylated by NAD^+^-dependent sirtuins (SIRT1 and SIRT3), with moderate activity against K220 and high activity against K211 and K212 in the HBD furin cleavage region. These sites of acetylation have not been previously reported, likely due to the peptide’s unique hydrophilic nature. Overall, our findings reveal that sirtuin-directed deacetylation of SOD3 restored furin cleavage, defining an important link between redox homeostasis and acetylation-directed metabolic regulation of extracellular oxidative stress.

## 1. Introduction

Extracellular superoxide dismutase (SOD3) is an antioxidant enzyme that scavenges superoxide, converting it to hydrogen peroxide. SOD3 is critically abundant in organs, including the lung and kidney [1], which are markedly susceptible to oxidative damage. Notably, SOD3 contains a heparin-binding domain (HBD) at its carboxyl terminus [2]. This region consists of a short sequence of basic amino acid residues (RKKRRR), giving it a high affinity for heparan sulfate proteoglycans and other components of the extracellular matrix (ECM). This polybasic region also acts as a recognition site for the proprotein convertase furin, and cleavage of the HBD after R215 can occur prior to SOD3 secretion. *In vivo*, the remaining basic residues, R210-R215, can be removed by an unknown carboxypeptidase, allowing for the complete removal of the HBD and redistributing SOD3 from tissues rich in type I collagen to the extracellular fluid [3]. The human single nucleotide polymorphism (rs1799895) found in SOD3 leads to an amino acid substitution of an arginine in the HBD to a glycine (R213G). This mutation reduces the affinity of this region for the ECM, leading to the redistribution of SOD3 into plasma and other extracellular fluids. For instance, the R213G variant increases SOD3 concentration in airway-lining fluids, making it protective in several pulmonary diseases, including emphysema [4], pneumonia [5], lung fibrosis [6], and asthma [7]. This variant also decreases SOD3 abundance in tissues, resulting in deleterious effects in cardiovascular diseases [8,9], revealing the importance of regulating the proteolytic removal of the HBD in protection against extracellular oxidative damage throughout the body. SOD3 is also implicated in other disease states where its deficiency induces activation of hepatic stellate cells, thereby increasing deposition of collagen in liver fibrosis [10], and its overexpression attenuates neural injury resulting from ischemic stroke [11].

**Fig. 1.**
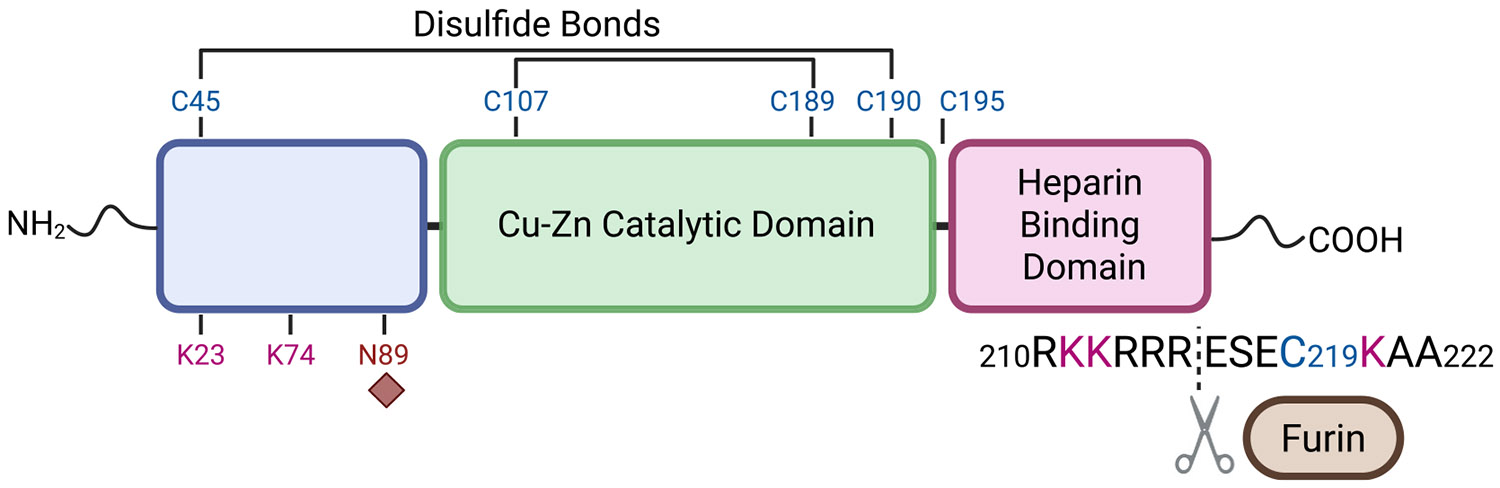
Domain substructure of SOD3. SOD3 contains an N-terminal signal peptide that marks it for excretion into the extracellular matrix. Prior to excretion SOD3 forms homotetramers, mainly through interactions between a loop near the N-terminus (residues 51–60). N-linked glycosylation on N89 improves SOD3 extracellular secretion and solubility. There exist two intra-subunit disulfide bonds in the active structure, between C45 and C190, and C107 and C189. Disulfide exchange with C195 forms the inactive form. The Cu–Zn catalytic domain ranges from residues 96–193. SOD3 has a heparin binding domain at its C-terminal end consisting of a region of polybasic amino acid residues (R210-R215) that can act as a recognition site for furin, which will cleave at the C-terminal side of R215. Prior to proteolytic processing C219 residues can bond together forming a homodimer.

SOD3 is regulated via disulfide bond formation for inter- and intramolecular structural dynamics, as well as furin cleavage site accessibility. Two intramolecular disulfide bridges are necessary for enzymatic activity (Fig. 1), and rearrangement of these disulfide bonds into a second bridge pattern renders SOD3 inactive [12]. The lability of these cysteine bonds points to a mechanism of redox regulation whereby disulfide exchange activates SOD3 in response to changes in the redox environment. In addition, SOD3 is regulated by an intermolecular disulfide with the carboxy-terminal cysteine (C219); this bond is required for homodimerization, and its reduction was found by Gottfredsen et al. [13] to increase susceptibility to furin cleavage. Dimers can form between SOD3 proteins with identical or distinct disulfide bridge patterns, and different combinations of these dimers, along with variabilities in the presence of the HBD, produce SOD3 tetramers with a range of activity and ECM affinity [12,13]. Aside from redox regulation, other post-translational modifications (PTMs) have been identified on SOD3, including C219 cysteinylation [13], the presence of which prevents homodimerization, and N89 glycosylation [14], which is required for SOD3 secretion.

Acetylation of lysine residues on histones has long been recognized as a key regulator of transcription. Still, the impact of this PTM on the function of non-histone proteins has been increasingly of interest as research uncovers the importance of acetylation in many physiological processes through its influence on protein structure, distribution, and activity [15]. For example, the cytosolic isoform of superoxide dismutase, SOD1, is acetylated at K71, resulting in decreased enzymatic activity [16]. Mapping of SOD1 location in the mouse nervous system also detected SOD1 K123 acetylation [17]. A fraction of SOD1 also accumulates in the mitochondria, and acyl-K123-mimicking mutants decrease SOD1 localization to the mitochondrial intermembrane space [18]. SOD2, the mitochondrial isoform, is found hyperacetylated on K68 and K122 in the liver with chronic alcohol use and in breast cancer cells [19]. Acetylation at these lysine residues was determined to inhibit enzymatic activity [20,21]. Interestingly, acetylation of SOD3 has remained uncharacterized, likely due to technical limitations with a non-targeted, MS-based approach. Our results show that, while acetylation does not change the enzymatic activity of SOD3, it significantly impacts proteolytic processing. We determined that furin cleavage of the HBD of SOD3 is blocked by lysine acetylation at the C-terminal basic region (R**KK**RRR), and that sirtuins, a class of NAD^+^-dependent deacetylating enzymes, deacetylate SOD3 with high specificity in this polybasic region. Remarkably, deacetylation of SOD3 by sirtuins restored its susceptibility to furin cleavage, thus regulating extracellular distribution.

Indeed, sirtuins are integral to the global antioxidant pathway [22], and our results indicate that the deacetylation of SOD3 plays a role in regulating extracellular redox status. Our *in vitro* analyses, utilizing a targeted MS-based approach, reveal that SOD3 is deacetylated by SIRT1 and SIRT3, which regulate furin processing. This occurs independently of the redox status of SOD3 and provides an additional mechanism for SOD3 regulation. Together, these findings present a novel approach to identifying SOD3 acetylation at a key regulatory site and provide an important connection between intracellular metabolic status and extracellular redox regulation via sirtuin activity.

## 2. Materials and methods

### 2.1. Reagents, recombinant protein, and antibodies

Reduced glutathione (GSH), oxidized glutathione (GSSG), acetyl coenzyme A (AcCoA) and 2-Mercaptoethanol (βME) were purchased from Sigma Aldrich (St. Louis, MO). Dithiothreitol (DTT) and sulfo–NHS–acetate (sNHSAc) were purchased from Thermo Fisher Scientific (Denver, CO). β-nicotinamide adenine dinucleotide hydrate (NAD^+^) was purchased from Cayman Chemical (Ann Arbor, MI). S-acetyl-l-glutathione (AcGSH) was purchased from Boc Sciences (Shirley, NY). Recombinant human SOD3 (#2122285) was purchased from MyBioSource (San Diego, CA). Anti-acetylated-lysine antibody (#9441) was purchased from Cell Signaling Technology (Danvers, MA). Horseradish peroxidase (HRP) (#PA1-28738) conjugated goat anti-rabbit IgG was purchased from Invitrogen (Carlsbad, CA). Recombinant human SIRT1 (#7714-DA-050) was purchased from R&D Systems (Minneapolis, MN), or expressed in-house, and percent purity as >95 % was demonstrated by SDS-PAGE. Recombinant human SIRT3 (#10011194) was purchased from Cayman Chemical. Furin (9450-47) was purchased from Peprotech (Cranbury, NJ) and reconstituted in 0.1 % BSA in DI water. pERTKR-AMC Fluorogenic Peptide Substrate (#ES013) was purchased from R&D Systems (Minneapolis, MN).

### 2.2. Purification of SIRT1

Cloning, expression, purification of the Human Sirtuin (SIRT1): The amino acid sequence of human encoding sirtuin protein (SIRT1) (Uniprot ID: Q96EB6) was synthesized from Twist Biosciences. The synthesized amino acid was cloned into a pET-Sumo vector having a purification histidine tag. The constructed plasmid was transformed into *Escherichia coli* BL21(DE3) cells. To express the protein, the cells were grown at 37 °C till the O.D.600 reached ≈0.6. Further, 0.25 mM of IPTG was added into the LB media and further flask was kept at 25 °C for overnight. The induced cells were harvested by centrifugation. The cell pellets were dissolved into lysis buffer and cells were lysed by repeating cycle of sonication. The protein was purified using Ni-NTA chromatography sumo-his tag was cleaved by ULP1 enzyme. Further pure protein was obtained by reverse nickel column. Purified protein was dialyzed several times into 50 mM sodium phosphate buffer, 20 mM NaCl, pH 7.0.

### 2.3. Acetylation

SOD3 (0.3 μg/μL) was incubated with sNHSAc at RT for 30 min in 20 mM sodium phosphate buffer pH 8.0. SOD3 was treated with 1 mM sNHSAc unless otherwise noted.

### 2.4. Deacetylation

Acetylated SOD3 (AcSOD3) (0.16 μg/μL) was incubated with SIRT1 (0.078 μg/μL) or SIRT3 (0.138 μg/μL), concentrations adjusted for purity, and 2 mM NAD^+^ in 1x SIRT assay buffer (#10010993 Cayman Chemical, Ann Arbor, MI) for 30 min at 37 °C. 4x Laemmli sample buffer containing βME was added to samples which were then heated at 90 °C for 5 min and loaded into a 12 % SDS-PAGE gel for immunoblot analysis or 12.5 % Criterion Tris-HCl protein gel for in-gel digestion (3450014) (Bio-Rad, Hercules, CA).

### 2.5. Activity assays

SOD3 activity was determined using an assay (S311-10) from Dojindo (Rockville, MD) per the manufacturer’s instructions. Acetylated and control samples were diluted to 0.005 μg/μL SOD3. Absorbance at 450 nm was measured at 1-min intervals for 20 min at 37 °C. All samples and controls were performed in triplicate and normalized to zero timepoints.

### 2.6. Immunoblotting

Samples were separated in a polyacrylamide gel in SDS buffer. Total protein abundance was determined using 2,2,2-trichloroethanol (TCE) and used to normalize the signal from the anti-acetylated-lysine antibody. Protein was semi-dry transferred to a polyvinylidene difluoride (PVDF, 0.45 μM) membrane in 10 mM Tris (base) and 192 mM glycine with 20 % (v/v) methanol at 150V for 1 h at 25 °C. Following transfer, membranes were blocked in 10 mM Tris-HCl, pH 7.8, 150 mM NaCl and 0.2 % (v/v) Tween-20 (TBS-T) containing 5 % (w/v) non-fat dry milk (blocking buffer) for 1 h at RT. Anti-acetylated-lysine antibody was diluted 1:1000 in blocking buffer and applied to membranes overnight at 4 °C. Membranes were then washed extensively in TBS-T followed by the addition of a HRP conjugated secondary antibody diluted 1:10,000 in blocking buffer for 1 h at RT. Blots were visualized using a Bio-Rad ChemiDoc MP Imaging Systems and analyzed using Bio-Rad ImageLab 6.1 software. Relative acetylation was calculated by normalizing SOD3 band chemiluminescence signals to the SOD3 total protein amounts using TCE band signals. Acetylation amounts were then normalized to the control sample.

### 2.7. Furin cleavage

Immediately following acetylation and/or deacetylation 0.079 μg/μL SOD3 sample was incubated with 0.004 μg/μL furin, 1 mM CaCl_2_, and 25 mM Tris-HCl pH 7.2 for 2 h or overnight at 37 °C. Cleaved samples were immediately examined by intact LC-MS/MS and immunoblotting. To examine how AcSOD3 redox state impacted furin cleavage, samples treated with 0.1 mM H_2_O_2_ or 1 mM βME were incubated in 25 mM Tris-HCl pH 7.2 at 37 °C for 1 h prior to furin cleavage. The sNHSAc was not removed from samples prior to incubation with furin and sNHSAc did not inhibit furin activity (Supp. 6). For each assay, 33 μL of Tris HCl pH 7.3, 37 μL NaPi pH 8.0, 10 μL of 10 mM CaCl2, 10 μL of furin±sNHSAc, and 10 μL of 500 μM pERTKR-AMC peptide were added to each well. A fluorescence microplate reader was used to acquire signal every 3 min at ex/em 380/460 nm and 37 °C for 45 min.

### 2.8. Intact protein analysis by LC-MS and LC-MS/MS

Sixteen μL of samples that underwent the furin cleavage protocol were chromatographically resolved on-line using a 2.1 × 50 mm, 5.0-μ PLRP-S 1000A column (Agilent Technologies) using a 1290 Infinity II LC system (Agilent Technologies). Mobile phases consisted of water +0.1 % formic acid (A) and 90 % aq. ACN +0.1 % formic acid (B). Samples were acquired using a flow rate of 0.2 ml/min using a gradient holding 5 % B for 2 min and 5–90 % B over 4 min for a total 6-min gradient. The gradient method was followed by a column wash at 90 % B for 2 min before returning to the initial condition over 2 min. Data were collected on a 6550 Q-TOF equipped with a dual jet stream source (Agilent Technologies) operated in Auto MS/MS and MS-only modes from 0 to 3.8 min and 3.8–12 min, respectively. MS/MS data were collected in positive-ion polarity over mass ranges 270–1700 m/z at a scan rate of 5 spectra/s for MS scans and mass ranges 50–1700 m/z at a scan rate of 3 spectra/s for MS/MS scans. All charge states were allowed, and precursors were sorted by abundance. Quadrupole isolation width was set to narrow and ramped collision energies were used with a slope of 3.1 and offset of 1 for charge states 1 and 2 and slope of 3.6 and offset of −4.8 for charge states 3 or higher. MS data were collected in positive-ion polarity over mass ranges 270–1700 m/z at a scan rate of 1.5 spectra/sec. Intact protein data was analyzed in MassHunter Qualitative Analysis version B.07.00 equipped with MassHunter Bioconfirm software (Agilent Technologies). The MS signal was averaged over the protein chromatographic peak and the average spectrum was deconvoluted using maximum entropy in MassHunter Bioconfirm software to determine the accurate mass of proteins present and determine if there was a shift from furin cleavage in furin positive samples compared to furin negative samples. To quantify furin cleavage percentages, manual integration of the deconvoluted spectra from 25,700–27,900 amu was performed and divided by the integration of 28,075–28,925 amu. This cleavage ratio for the AcSOD3 no-furin (negative control) sample was subtracted from the cleavage ratio for each treatment and normalized to the SOD3 +furin (positive control) sample. Furin peptide cleavage products MS/MS spectra were manually annotated and identified using UCSF ProteinProspector and the MS-Product peptide utility program version 6.6.6. The formula for these identified peptides were then used in the find by formula algorithm in MassHunter Qualitative Analysis to quantify the peptide area signal in each sample with a height cut-off of 10,000 counts and mass error of ±10 ppm.

### 2.9. Acetyl peptide quantification by LC-MS/MS

SOD3 control, acetylated, acetylated plus Sirt1, and acetylated plus Sirt 3 samples were prepared and analyzed using LCMS methods similar to a previous publication [23]. Samples were processed and digested following the Preomics iST Kit protocol. 2 μL of samples were aliquoted to 1.5 mL microfuge tubes. 50 μL of lysis buffer was added and samples were heated at 90 °C for 10 min for reduction and alkylation. 50 μL of provided trypsin was added to each sample and reacted for 2 h at 37 °C. Sample digestion was stopped, washed twice, and eluted using buffers provided with the kit. Final eluents were dried in a speedvac at 45 °C. Samples were re-suspended in 12 μL of 2 % acetonitrile +0.1 % formic acid. 2 μL of Samples were loaded onto a 2 cm PepMap 100, nanoviper trapping column and chromatographically resolved online using a 0.075 × 250 mm, 3.0 μm EASY-Spray PepMap RSLC C18 reverse phase nano column (Thermo Scientific) using an Ultimate 3000 RSCLnano LC system (Thermo Scientific). Mobile phases consisted of water +0.1 % formic acid (A) and 100 % acetonitrile + 0.1 % formic acid (B). Samples were loaded onto the trapping column at 5.0 μL/min for 3.2 min at initial condition before being chromatographically separated at a flow rate of 300 nl/min using a gradient of 3–33 % B over 15.8 min at 40 °C. This shortened trapping period was necessary to prevent our target peptide (RKacKacRRR) from being washed off the trapping column and preventing detection. The gradient method was followed by a column wash at 70 % B for 5 min. Data were collected on an Orbitrap Eclipse (Thermo Scientific) operated using intensity-dependent CID MS/MS to generate peptide ID’s in experiment 1 and a parallel reaction monitoring (PRM) method was used to target the KacKacR peptide (258.1687 m/z, z = 2) in experiment 2, a necessary step for the thorough identification of this acetylated peptide. Experiment 1 settings were as follows: MS1 spectra were acquired in the Orbitrap (Resolution = 60k; AGC target = 100 %; MaxIT = Auto; RF Lens = 30 %; mass range = 150–1600; Profile data). Precursors selected for MS/MS were filtered by MIPS model set to peptide with an intensity threshold of 15000 and only charge states 2–6 were allowed. Dynamic exclusion was employed for 8 s. MS2 spectra were collected using CID in the Orbitrap (Isolation window = 1.2 m/z [quadrupole], Resolution = 7.5k; AGC target = 100 %; MaxIT (ms) = 11; CID = 35 %). Experiment 2 was deployed from 10 to 11.3 min, to detect elution of the acetylated HBD peptide. The resolution was set at 120k, the AGC target at 50,000, max fill time of 246 ms, the quadrupole isolation at 1 m/z, and a normalized collision energy of 35.

Experiment 1 data were searched and extracted using SEQUEST HT and the label-free quantitation workflow in Proteome Discover software version 2.5.0.400 utilizing the minora feature detector, feature mapper and precursor ions quantifier algorithms. Spectra were searched against a FASTA file containing human SOD3, SIRT1, and SIRT3 SwissProt sequences allowing up to 4 missed tryptic cleavages with fixed carbamidomethyl (C) and dynamic oxidation (M) and acetylation (K) modifications. The monoisotopic peptide mass tolerance allowed was ±10.0 ppm and the MS/MS tolerance was ±0.025 da. Peptides were adjusted to a 1 % false discovery rate (FDR) using the percolator algorithm and only high-confidence peptides were quantified. A signal to noise (S/N) threshold of 3 was set for minora feature detector and peptide spectrum match (PSM) confidence levels were set to high for feature ID linking. A coarse retention time (RT) alignment of data was performed using default settings. Precursor quantification was based on ion intensity. ESECKacAALE and KacKacR peptide MS/MS spectra were manually annotated and identified using UCSF ProteinProspector and the MS-Product peptide utility program. Extracted ion chromatograms with a mass error of 5 ppm were produced in Freestyle software (Thermo Scientific) at *m/z* of 539.7397 da in MS1 scans for the ESECKacAALE peptide and *m/z* of 175.1190 da corresponding to the most abundant fragment ion (y1 ion) in the targeted PRM experiment for the KacKacR peptide. The intensity value of each peak was then recorded and used for quantitation between samples. All peptide intensity values were normalized to total SOD3 protein signal within each sample by summing all identified and quantified peptides in the proteome discoverer label-free quantitation workflow before fold change calculations were made between samples.

### 2.10. Statistical analysis and illustrations

For immunoblot densitometry, graphs represent the within treatment group average (n = 3, per group) with error bars denoting standard deviation. The statistical significance of the difference for SIRT1 and SIRT3 deacetylation was assessed by a one-way ANOVA with Dunnett’s multiple comparison test. Statistical analyses and graphical representations were done in GraphPad Prism (10.4.1). Figure schematics were made in Biorender.com.

## 3. Results

### 3.1. SOD3 activity is not impacted by acetylation

**Fig. 2.**
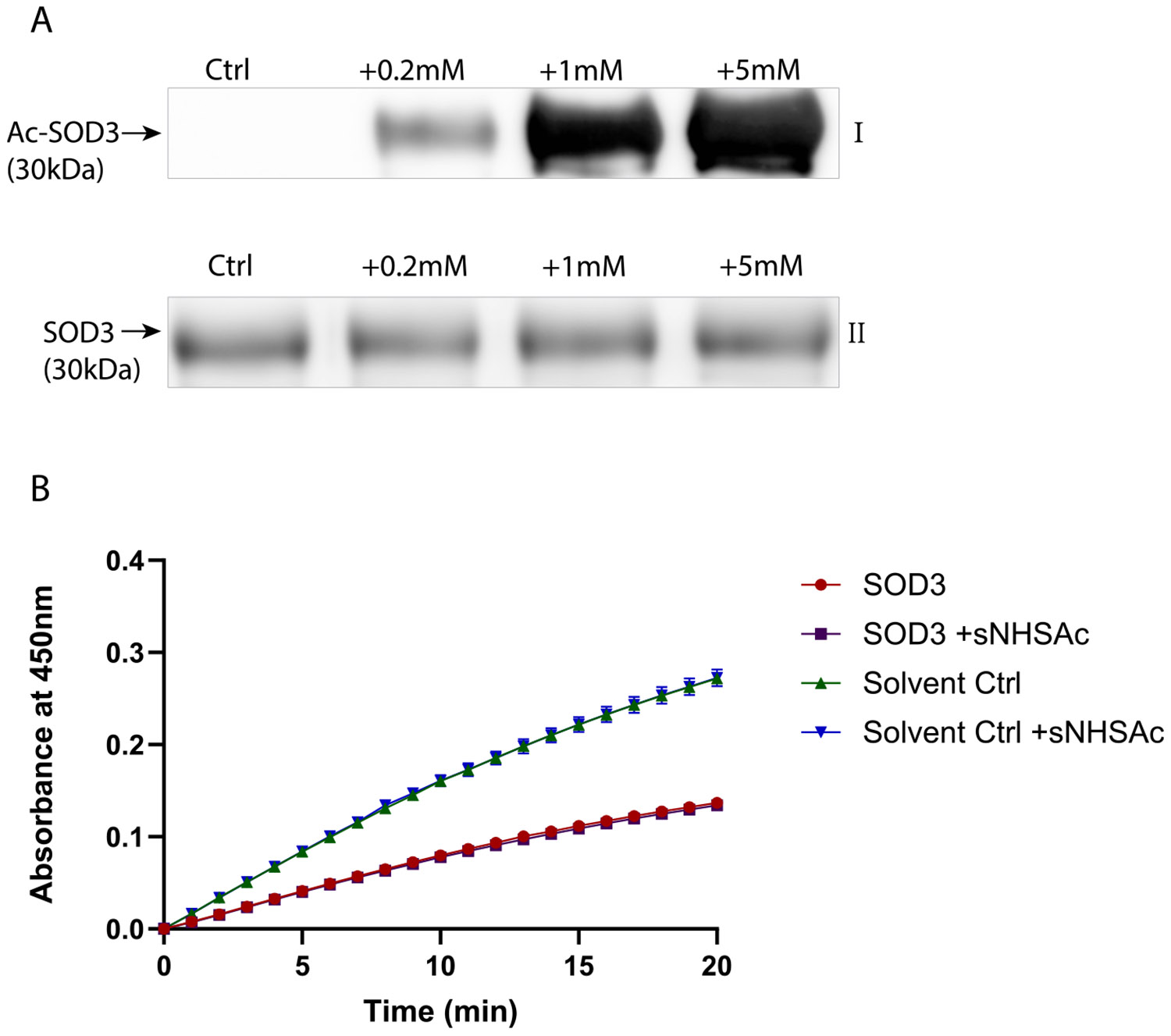
SOD3 activity is not affected by acetylation. (A) Treatment with 0.2–5 mM sNHSAc for 30 min at RT dose-dependently acetylated recombinant SOD3 as evaluated by (I) anti-acetylated-lysine immunoblotting. (II) TCE labeling was used to monitor protein loading and transfer. (B) No change was observed in SOD3 activity when treated with 1 mM sNHSAc. Activity was measured by the decrease in the rate of absorbance of WST-1 formazan at 450 nm with the addition of SOD3. Consumption of superoxide by SOD3 inhibits the reduction of WST-1, preventing WST-1 formazan formation. Error bars depict the standard deviation (n = 3).

Since lysine acylation is known to alter the enzymatic activity of SOD1 [16,24] and SOD2 [20,21], we evaluated the effect of acetylation on the activity of SOD3. To determine how lysine acetylation impacts activity, SOD3 was dose-dependently acetylated by sNHSAc and monitored by immunoblotting (Fig. 2A), and a concentration of 1 mM sNHSAc was chosen for all subsequent acetylation reactions. sNHSAc is a less reactive, but more selective modifier of lysine amines than the commonly used acetylating reagent acetic anhydride [25,26]. The physiologically relevant reagents, S-acetyl glutathione (AcGSH) and acetyl coenzyme A (AcCoA), were also found to acetylate SOD3 successfully (Supp. 1) but were not used in further experiments as these reagents may also modify cysteine residues [13]. For instance, Gottfredsen et al. [10] showed that increasing concentrations of reduc- ed/oxidized glutathione prevented SOD3 dimerization, which could confound experimental interpretations of SOD3 acetylation using AcGSH. Similarly, CoA has been found to modify cysteine residues [27]. Interestingly, acetylation of SOD3 did not significantly alter its activity (Fig. 2B). While the activity of many non-histone proteins can be affected by lysine acetylation, the insensitivity of SOD3 activity to acetylation is unsurprising as none of the lysine residues are located within the catalytic domain as determined by our site-specific MS analysis, in contrast to the known acetylated residues of SOD1 and SOD2.

### 3.2. Proteolytic processing of SOD3 by furin is blocked by acetylation

**Fig. 3.**
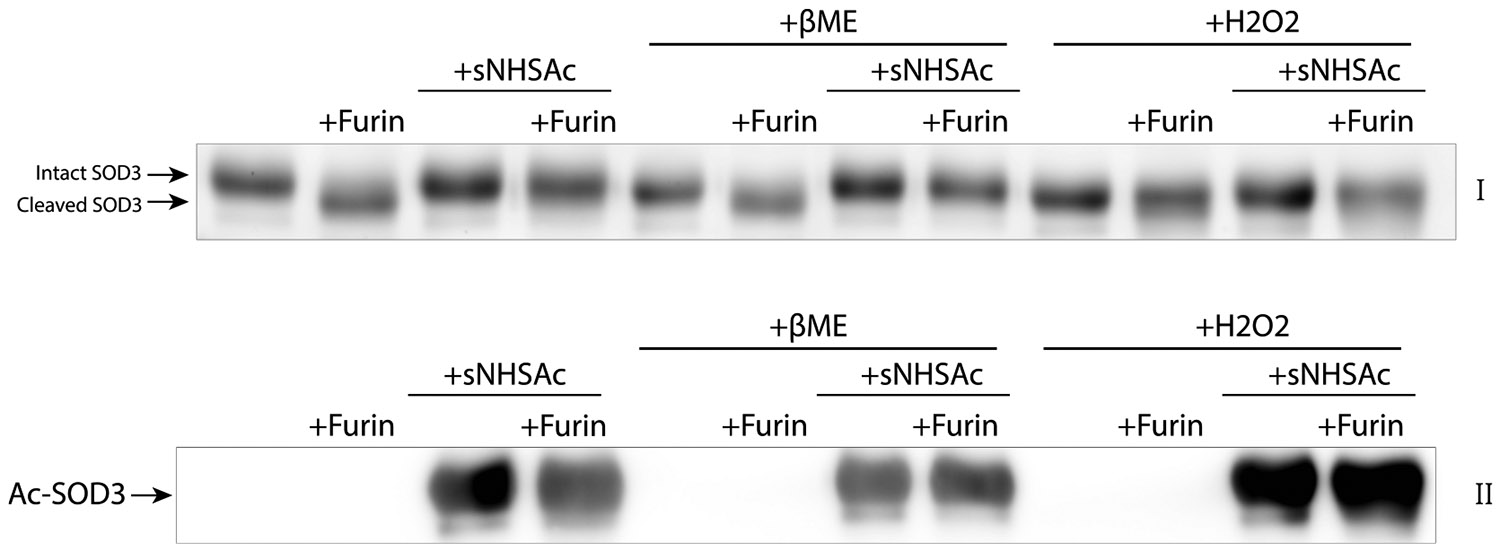
Furin cleavage of SOD3 is blocked by acetylation. Treatment of SOD3 and AcSOD3 with 0.1 mM H_2_O_2_ or 1 mM βME followed by incubation with furin overnight at 37 °C was performed to evaluate the impacts of reduction and oxidation on furin cleavage. Proteolysis was monitored by electrophoretic mobility in a reducing Western blot visualized by TCE labeling (I), the uncropped blot with molecular weight markers is included in Supp. Fig. 2A. Neither H_2_O_2_ nor βME affected furin cleavage or the prevention of cleavage by acetylation. Anti-acetyl-lysine immunoblotting confirmed acetylation of SOD3 (II).

Given the complex regulatory nature of SOD3, we sought to examine how lysine acetylation impacted the polybasic, furin-recognition, C-terminal region. The removal of the HBD by furin was analyzed by immunoblotting and validated by mass spectrometry. Cleavage was seen as a change in electrophoretic mobility in SDS-PAGE corresponding to a ~1 kDa mass loss from the protein (Fig. 3) and by mass spectrometry as a shift in molecular weight of the intact protein along with MS/MS spectra of cleaved peptides (Fig. 5B, Supp. 5). Acetylated SOD3 did not exhibit this mass shift demonstrating that acetylation blocks furin cleavage of SOD3.

### 3.3. Effect of reduction and oxidation on SOD3 cleavage

To evaluate the impact of SOD3 dimerization and disulfide status on lysine acetylation and furin cleavage, the experiments above were repeated in the presence of hydrogen peroxide (H_2_O_2_) or the reducing reagent, β-mercaptoethanol (βME). Neither reduction nor oxidation of SOD3 impacted furin cleavage or the ability of lysine acetylation to inhibit furin cleavage (Fig. 3). C219, which is responsible for SOD3 homodimerization, is adjacent to K220; however, results utilizing reducing and oxidizing conditions indicate that the mechanism of blockage of furin cleavage by acetylation is independent of SOD3 dimerization. While SOD3 is known to be inactivated through cleavage by H_2_O_2_ at P112, the concentration of H_2_O_2_ used here, 0.1 mM, is not sufficient to induce oxidative fragmentation and inhibit SOD3 [28]. These experiments define SOD3 as an enzyme regulated independently by metabolically linked acetylation and through oxidative stress-induced thiol redox changes.

### 3.4. SOD3 is deacetylated by sirtuins

**Fig. 4.**
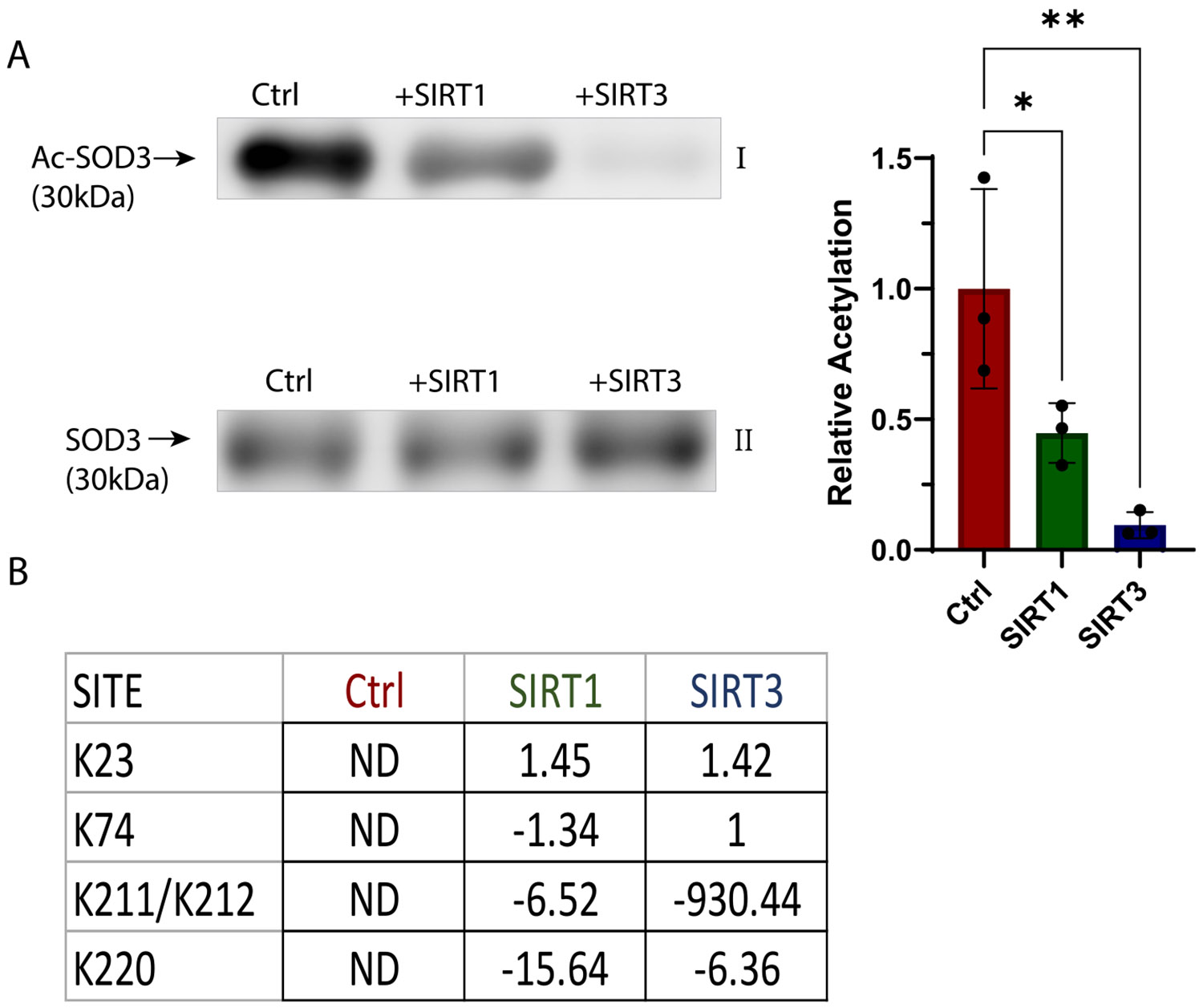
SIRT1 and SIRT3 deacetylate SOD3. Acetylated SOD3 was incubated with SIRT1 or SIRT3 and 2 mM NAD + for 30 min at 37 °C. Global deacetylation of SOD3 was evaluated by anti-acetylated-lysine immunoblotting (AI) and quantified by densitometry with normalization to total protein visualized with TCE labeling (AII). Data are expressed as mean with SD; a one-way ANOVA was performed on triplicates *p *<* 0.05, **p *<* 0.01. Site specific deacetylation was quantified by proteomics of trypsin digests in terms of fold change compared to acetylated SOD3 (B). ND indicates that acetylation at this site was not found in Ctrl SOD3.

Sirtuin-directed deacetylation of SOD1 and SOD2 is crucial in regulating cytosolic and mitochondrial oxidative stress [20,24]. To determine if SOD3 is also a target of sirtuins, acetylated SOD3 was incubated with sirtuins 1, 2, and 3. Immunoblotting was used to quantify global deacetylation, and SOD3 was found to be deacetylated at 55 % and 91 % by SIRT1 and SIRT3, respectively (Fig. 4A). Based on our analysis, SIRT2 displayed no activity toward AcSOD3 and was not examined further (data not shown). The deacetylated lysine residues were determined by mass spectrometry analysis of trypsin-digested samples (Fig. 4B). SIRT3 had high specificity to lysine residues K211 and K212 in the polybasic region of the HBD, with a 930-fold decrease in acetylated peptide versus AcSOD3. SIRT1 also deacetylated these residues, exhibiting a 6.52-fold decrease, but had greater selectivity to the C-terminal K220 (15.64-fold decrease). Our results include spectra of the KacKacR and ESECKacALLE peptides (Supp. 3, 4). The KacKacR peptide uniquely arises from acetylation of the furin target site R**KK**RRR and trypsin cleavage, and our MS/MS analyses confirm this tripeptide is K211-R213 from recombinant AcSOD3. Lastly, sirtuins did not have any notable deacetylation activity toward K23 or K74.

### 3.5. Sirtuins restore furin-mediated removal of SOD3’s HBD

**Fig. 5.**
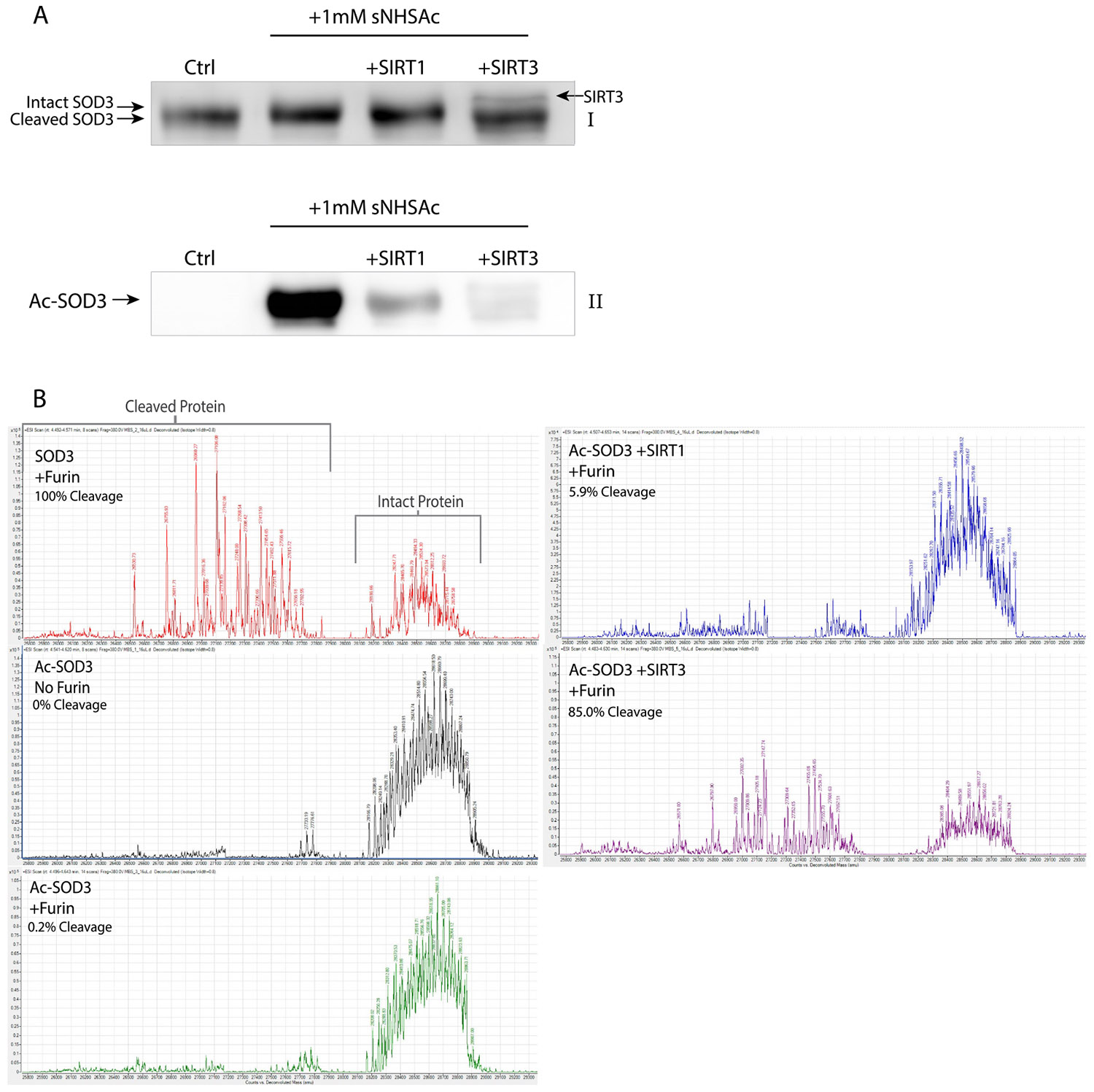
Deacetylation of SOD3 restores furin cleavage. SOD3 acetylated with 1 mM sNHSAc and then deacetylated by SIRT1 or SIRT3 was incubated with furin overnight at 37 °C. All samples shown in (A) are incubated with furin, acetylation blocks furin cleavage as seen by a change in electrophoretic mobility in SDS-PAGE, and SIRT3 restores this shift (AI), the uncropped blot is included in Supp. Fig. 2B. SIRT3 has an observed MW of 33.5 kDa and can be seen as a band above cleaved SOD3. Anti-acetylated-lysine immunoblotting confirms acetylation and deacetylation of these samples (AII). The shift in SOD3 mass was also evaluated by intact mass spectrometry, integration of the deconvoluted spectra from 25700 to 27900 amu was performed and divided by the integration of 28075–28925 amu. This cleavage ratio for the AcSOD3 no-furin sample was subtracted from the cleavage ratio for each treatment and normalized to the SOD3 +furin sample. Percent cleavage is therefore relative to defining the SOD3 + furin cleavage ratio as 100 % or complete cleavage. SIRT1 restored 5.9 % of furin cleavage and SIRT3 restored 85.0 % (B).

Given that removal of the HBD is blocked by acetylation, and sirtuins deacetylate SOD3, we hypothesized that deacetylation by SIRT1 and SIRT3 could restore cleavage. Further, the differences in lysine site-specificity between SIRT1 and SIRT3 would also provide mechanistic insight into the key regulatory residues. To test this, SOD3 deacetylated by SIRT1 or SIRT3 was incubated with furin. Labeling with trichloroethanol showed SOD3 incubated with SIRT3 and furin to have similar mobility in SDS-PAGE to the SOD3 + furin control, indicating that SIRT3 deacetylation of SOD3 restores furin cleavage (Fig. 5A). The change in acetylation between samples is not sufficient in and of itself to shift the electrophoretic mobility of SOD3 in a reducing gel, demonstrating that this downward shift of SOD3 is indeed due to loss of the ~1 kDa C-terminal end of the protein. The SDS-PAGE results were confirmed by intact protein mass spectrometry analyses and cleavage was seen by a ~1 kDa shift in mass of the intact protein (Fig. 5B), as well as by the appearance of HBD peptide fragments (Supp. 5A). SOD3 deacetylated by SIRT1 exhibited 5.9 % of the cleavage detected in the SOD3 + furin control, and SOD3 deacetylated by SIRT3 robustly restored removal of the HBD by furin with 85.0 % cleavage. The intact SOD3 spectra revealed a multitude of peaks due to the presence of many unique SOD3 proteoforms; hence, acetylation was difficult to resolve by mass spectrometry. Therefore, anti-acetylated lysine immunoblots were utilized to confirm successful acetylation with sNHSAc (Fig. 5). Cleavage of SOD3 by furin is thought to occur at the amino acid R215, as the R213G SOD3 variant can still undergo this initial cleavage event [2]. Proteolysis by furin C-terminally of R215 is confirmed by our MS/MS spectra of the cleavage products. We identified a peptide fragment from E216 to the C-terminus with the C219 disulfide still intact and forming a peptide dimer (Supp. 5B). This peptide was also found with cysteinylation on C219, which is a previously reported SOD3 PTM [13] (Supp. 5C). This version of recombinant SOD3 from MyBioSource includes leucine and glutamic acid at the C-terminus, therefore all peptides found contained the amino acid sequences ESECKAALE.

## 4. Discussion

Considering the role of SOD3 in redox signaling and control, this research demonstrates a significant advancement in our understanding of SOD3 regulation and an expansion of the targets by which sirtuins mediate extracellular antioxidant responses. Chronic oxidative distress and inflammation can lead to fibrotic diseases, and SOD3 is influential in both the development and resolution stages of fibrosis. For example, administering recombinant SOD3 to mice during the progression period of fibrosis improves lung architecture and function [29]. Sirtuins are also involved in fibrosis. For example, SIRT1 regulates the TGFβ signaling pathway by deacetylating the p65 subunit of NFκB^30^ and interacting with p300 [30] and Smad [31]. SIRT3 overexpression also ameliorates fibrosis by inhibiting myofibroblast differentiation [32]. Notably, the deacetylation of SOD2 is predicted to be a major mechanism through which SIRT3 reduces mitochondrial DNA damage and apoptosis in lung fibrosis [33]. The impact of acetylation on SOD3 found herein may likewise be of critical importance in liver fibrosis. For example, hepatic stellate cells in mice with SOD3 knockout exhibited increased production of collagen type I and α-smooth muscle actin, leading to enhanced fibrosis. Interestingly, SIRT1 expression was downregulated in these mice, and SIRT1 overexpression ameliorated the exacerbation of damage caused by SOD3 deficiency [10].

Acylation of SOD1 and SOD2, as well as their interactions with sirtuins, has been thoroughly investigated. SOD1 acetylation at K71 disrupts binding with the copper chaperone (CSS), which provides SOD1 with the copper necessary for catalysis [16]. SIRT1 removes acetylation at this lysine residue, thereby restoring the activity of SOD1. Overexpression of SIRT1 even extended the lifespan of mice with amyotrophic lateral sclerosis (ALS) associated with SOD1 mutation [34]. SOD1 has also been revealed as a target of SIRT5, which desuccinylates K123 [24]. Acetylation at K68 in the manganese catalytic domain of SOD2 inhibits its activity. Nutrient starvation and increased mitochondrial ROS induce SIRT3, which deacetylates SOD2, reactivating it and helping restore cellular antioxidant capacity and homeostasis [20]. SOD2 is also inhibited by acetylation at K122, and ethanol consumption results in elevated acetylation at both K122 and K68 [21]. Surprisingly, mice with knock-in of a SOD2 mutation mimicking constitutive deacetylation of K68 (SOD2^K68R^) developed cardiomyopathy, revealing the value of adaptive modulation of acetylation at key residues [35]. However, there remains a dearth of information about acylation PTMs on SOD3. N-glycosylation is one of the few SOD3 PTMs to be studied. Ota et al. [36] determined the structure of the glycan on N89, and they showed that CHO cells, deficient in the machinery necessary for glycan maturation, secreted less SOD3 and produced a lower ratio of the cleaved form. Work by Gottfredsen et al. [13] on SOD3 redox regulation discovered cysteinylation of C219 of the SOD3 monomer, where this PTM prevented intermolecular disulfide formation. To evaluate whether the effects of acetylation of SOD3 were dependent upon a thiol redox mechanism at C219, SOD3 was treated with H_2_O_2_ or βME before incubation with furin. Still, neither treatment impacted furin cleavage or its prevention by acetylation. H_2_O_2_ has been shown to induce furin cleavage in cells [13]; however, our results suggest that this is a possible downstream consequence of redox signaling, rather than a direct interaction between H_2_O_2_ and SOD3 or furin. Furin cleaves at the C-terminus end of the consensus site RX(K/R)R and given furin’s affinity for polybasic regions we hypothesize that masking of the positive charge of lysine residues in the HBD by acetylation will shield this region from furin recognition [37]. The acetyl moiety may also sterically hinder the access of furin to the RKKRRR region on SOD3. Overall, our characterization of lysine deacetylation of SOD3 revealed it as a target of both SIRT1 and SIRT3.

Sirtuins act as both metabolic sensors and coordinators of responses to oxidative stress. SIRT1 is induced during fasting and regulates proteins in catabolic ATP-producing pathways. Moreover, SIRT1 is bidirectionally connected with AMPK, a cellular energy sensor [38], and deacetylates PPAR-γ [39] and PGC-1α [40], regulators of gluconeogenesis and fatty acid oxidation. As to its role in antioxidant responses, SIRT1 protects from H_2_O_2_-induced apoptosis by increasing catalase [41] and SOD2 expression. SIRT3 is also a coordinator of metabolic and antioxidant pathways through its deacetylation of FOXO3 [42] and other enzymes involved in fatty acid oxidation and response to oxidative stress [43,44]. In this study, SIRT1, which shuttles between the nucleus and cytoplasm, and SIRT3, which is localized to the mitochondria, were both found to deacetylate SOD3, reflecting another example of substrate redundancy within the sirtuin family [45]. The *in-vivo* interaction between SOD3 and sirtuins remains to be evaluated and is a limitation of the current study. A number of examples exist supporting sirtuin activity toward proteins across subcellular compartments. Indeed, SOD1 is a cytosolic superoxide dismutase that has been reported to be regulated through SIRT5 desuccinylation, a known mitochondrial desuccinylase [24]. Therefore, a similar interaction may occur between SOD3 and SIRT3. Future *in vivo* studies will aim to determine the effect of SIRT1 and SIRT3 overexpression or knockdown on SOD3 acetylation and extracellular distribution, through regulation of HBD cleavage.

Our *in vitro* analysis of SOD3 acetylation provides a blueprint for detecting acetyl-SOD3 *in vivo*. The regulatory acetyl-lysines that interfere with furin recognition of SOD3 is extremely hydrophilic, which necessitates a shorter trapping duration for chromatography. Standard LC-MS chromatography methods would prevent retention and detection of this acetyl-peptide. The PRM method is also required since it allows for high quality spectra with peptide fragmentation across the entire elution period, rather than a data-dependent acquisition (DDA) approach, which can generate low quality fragmentation and prevent identification. These technical factors likely contribute to the lack of acetyl-SOD3 reported in the literature. Our ongoing investigation will explore the regulatory nature of SOD3 acetylation in murine models of hepatic and cardiovascular disease. Indeed, the regulation of SOD3 by sirtuins represents a critical link between cellular redox and metabolic homeostasis, which has significant implications in the pathogenesis of a myriad of diseases, particularly regarding extracellular antioxidant capacity. Our thorough site-specific analyses of SOD3 lysine acetylation by immunoblotting and mass spectrometry revealed the high selectivity of SIRT3 for the lysine residues within the HBD, showing that these are key residues preventing furin cleavage of acetylated SOD3.

## Supplementary Material

1

## Appendix A. Supplementary data

Supplementary data to this article can be found online at https://doi.org/10.1016/j.rbc.2025.100062.
