# Supplementary material for: Deacetylation of SOD3 by sirtuins restores furin cleavage
Source: Redox Biochem Chem. Author manuscript; Available in PMC 2026 Jan 7. (PMC12774472; doi:10.1016/j.rbc.2025.100062)
Supplement: 1 [file NIHMS2129735-supplement-1.zip › 250930 SOD3 Furin Revisions clean1_V2.doc]

**Supplemental 1.** SOD3 is dose-dependently acetylated by AcGSH (A) and AcCoA (B). SOD3 was incubated with 1-5 mM AcGSH in 20 mM sodium phosphate buffer pH 8.0 at 37 °C for 1 hr. For acetylation by AcCoA 0.3ug/uL SOD3 was incubated with 5 mM or 20 mM AcCoA in 90 mM Tris-HCl pH 8.0, and 270 mM NaCl at 37 °C for 3 hrs. Bands shown are monomers from nonreducing western blot visualized with TCE (AI, BI) and anti-acetyl-lysine immunoblotting (AII, BII).

**Supplemental 2.** Uncropped western blot images showing the change in mobility of SOD3 with furin cleavage. (A) corresponds to the western blot shown in Fig. 3I, and (B) to that in Fig. 5AI. Molecular weight markers from BioRad Precision Plus Protein Standards are labeled.

**Supplemental 3.** MS^2^ spectra of K(Ac)K(Ac)R peptide from tryptic digest of acetylated SOD3.

**Supplemental 4.** MS^2^ spectra of ESCK(Ac)AALE peptide from tryptic digest of acetylated SOD3

**Supplemental 5.** Extracted ion chromatograms of peptides resulting from furin cleavage of SOD3. The table includes all the peptides searched for these samples, none of these peptides were found in the SOD3 alone, AcSOD3 + furin, or AcSOD3 +SIRT1 + furin samples (A). In the detected peptides C219, which is responsible for SOD3 dimerization, forms a disulfide with a C219 in another peptide (B) or with a free cysteine amino acid (C). Annotated MS^2^ of these peptides confirms their identity and that furin cleaves after R215.

**Supplemental 6. The acetylating agent sNHSAc does not inhibit furin.** Furin was incubated with 0.526mM sNHSAc, which is greater than twice the concentration furin is exposed to in the SOD3 cleavage assays. Furin activity was measured by the rate of fluorescence measured at an excitation/emission of 380/460nm. Error bars depict the standard deviation (n=3).
